# Confluent and Reticulated Papillomatosis of Gougerot-Carteaud on Black Skin: Two Observations

**DOI:** 10.1155/2016/2507542

**Published:** 2016-04-05

**Authors:** Kouadio Celestin Ahogo, Patrice Ildevert Gbery, Vagamon Bamba, Yao Isidore Kouassi, Elidje Joseph Ecra, Kouame Alesandre Kouassi, Ange Sylvain Allou

**Affiliations:** ^1^Department of Dermatology and Venerology, Teaching Hospital of Treichville, BP 3, Abidjan, Côte d'Ivoire; ^2^Department of Dermatology and Venerology, Teaching Hospital of Bouaké, Bouaké, Côte d'Ivoire

## Abstract

Confluent and reticulated papillomatosis of Goujerot-Carteaud is a rare and benign skin disease characterized by flat papules taking a reticulated appearance. It is a skin disease of unknown etiology and nosology that is always discussed. This disease preferentially involves the chest and interscapular regions. It is a condition probably underdiagnosed in black skin because it generally simulates a pigmented tinea versicolor. This pathology withstands antifungal treatment but has a particular sensitivity to cyclines thus constituting a distinguishing criterion, useful for diagnosis which should be evoked in front of these reticulated confluent papules.

## 1. Introduction

Confluent and reticulated papillomatosis of Gougerot-Carteaud (CRPGC) is a rare dermatosis characterized by asymptomatic small, flat, and keratosic papules. It is a skin disease of unknown etiology and nosology that is always discussed [1]. It was considered as a clinical form of acanthosis nigricans or amylose [2]. This is a disease underdiagnosed especially in black skin and it is very often confused with a pigmented form of tinea versicolor. We report two cases.

## 2. Observation 1

**Figure 1:**
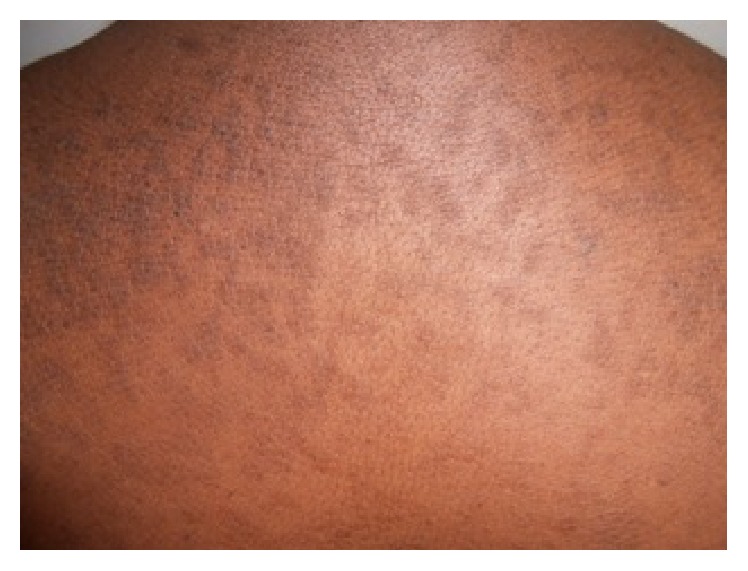
Large losangic placards of the upper back.

**Figure 2:**
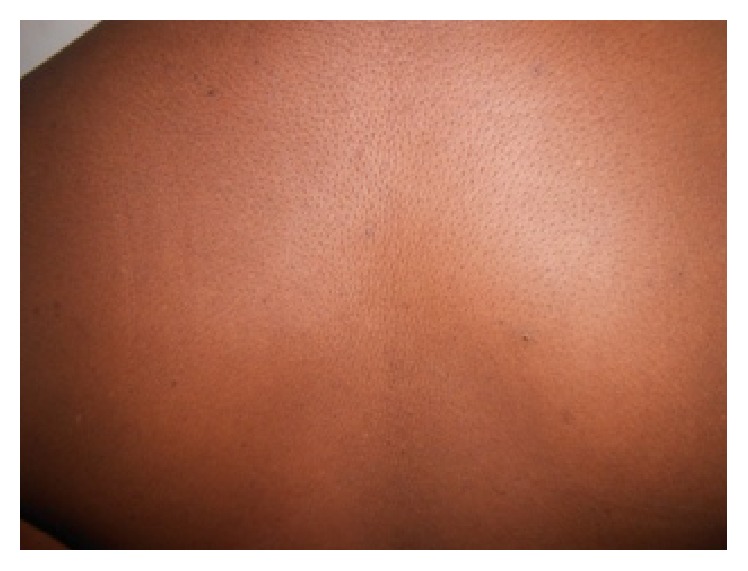
Complete cure of the patient after two weeks.

A 38-year-old patient with no particular antecedents presented for more than six months with flat papular lesions 1–5 mm in diameter, grayish pigmented color, and verrucose surface. These lesions began on the interscapular area to extend to the entire chest region and upper back. Then they came together in places by large losangic placards at the thoracic region (Figure 1). The lesions were neither painful nor itchy. The diagnosis of tinea versicolor pigmented form was retained many times in dermatology consultation. During its therapeutic itinerary all local and systemic antifungal treatments by different families have remained ineffective. In paraclinical assessment, mycological samples could not keep germ and histological examination obtained from the lesional skin revealed hyperkeratosis, acanthosis, and papillomatosis. It was not specific. Faced with these networked, confluent flat papules and this unspecific paraclinical assessment, diagnosis of confluent and reticulated papillomatosis of Gougerot-Carteaud was secondarily mentioned. Treatment with minocycline at a dose of 100 mg/day resulted in complete cure of the patient in two weeks (Figure 2).

There was no recurrence after six months of regress.

## 3. Observation 2

**Figure 3:**
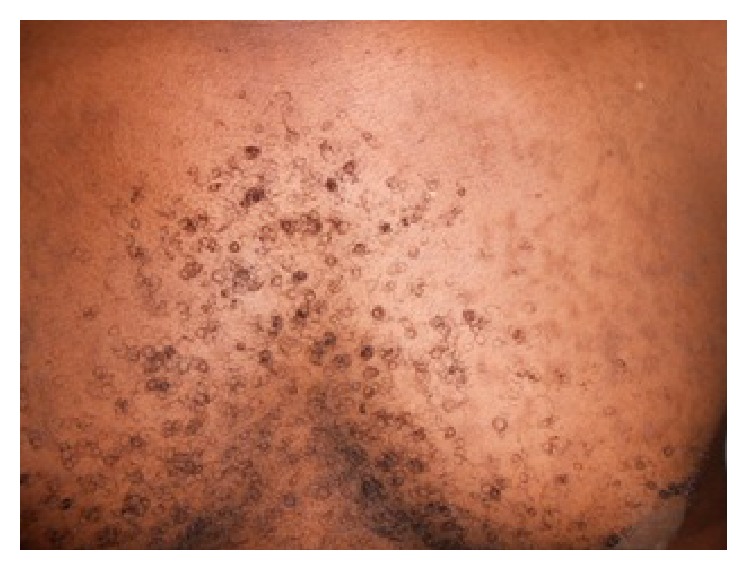
Flat papular lesions in the pectoral region.

**Figure 4:**
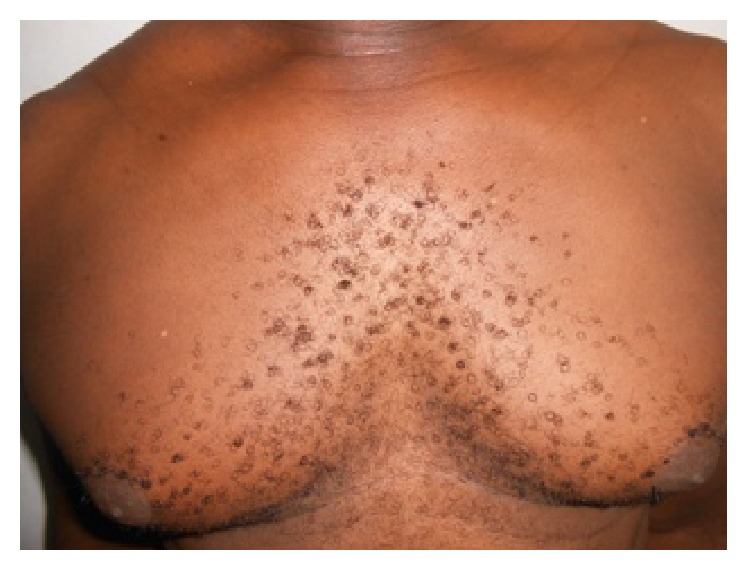
Complete recovery of the patient after 15 days.

Mr. BK 29 years old, with a history of multiple episodes of chlamydial urethritis, had seven-month flat papular lesions and pigmented slightly squamous surface. These lesions sat in the pectoral region and upper back (Figure 3). They were without functional signs. The diagnosis of tinea versicolor pigmented form was also mentioned several times. All antifungal treatments were ineffective, but the patient noted a disappearance of cutaneous lesions after each treatment of its episodes of urethritis with doxycycline. He used to take doxycycline for 10 days at a dose of 200 mg/day. However this healing was always followed by recurrence approximately one month later. In paraclinical assessment, mycological samples taken could not keep germs. Histological examination of a skin biopsy room was noncontributory. Faced with this unspecific paraclinical assessment of these network papules, we discussed the diagnosis of confluent and reticulated papillomatosis of Gougerot-Carteaud and instituted treatment with minocycline at a dose of 100 mg/day. This therapy resulted in complete cure of the patient in 15 days (Figure 4). No recurrence was noted after nine months of regress.

## 4. Discussion

Confluent and reticulated papillomatosis of Gougerot-Carteaud is a rare and benign dermatosis of the young subject; it characterized by flat pigmented, hyperkeratotic squamous papules. These confluent lesions take generally a reticulated appearance. They have little or no pruritic. The cause is not known: keratinization disorder, abnormal response of the individual follicular bacteria, or Malassezia [3]. The disease is preferentially seated in inter mammary and interscapular regions with potential extension to other sites. The involvement of the face is exceptional [4]. Confluent and reticulated papillomatosis of Gougerot-Carteaud is rare and probably underdiagnosed because these lesions often have a clinical aspect simulating tinea versicolor [5]. Thus the diagnosis of tinea versicolor pigmented form is most often brought on black skin. The paraclinical assessment of the condition is poor. Mycological samples are negative and the histology is not specific in general. Histopathological examination of a punch biopsy obtained from the lesional skin reveals hyperkeratosis, acanthosis, and papillomatosis, with scant perivascular lymphocytic infiltration. Periodic acid-Schiff staining demonstrates no fungal cells [6]. Therapeutic measures are many and varied due to pathogenic unknowns: antimycotics, oral retinoids, derivatives of vitamin A, and derivatives of vitamin D. However, this pathology has a special sensitivity to tetracyclines. Even response to minocycline is more reported in the literature; doxycycline seems to be as efficient as minocycline, with less risk of drug reaction with eosinophilia and systemic symptoms, especially in black skin patients.

Davis et al. [7] proposed the diagnostic criteria for confluent and reticulated papillomatosis of Gougerot-Carteaud on the basis of a study on 39 patients as follows: (i) clinical findings of scaly brown macules and patches, with at least some appearing reticulated and papillomatous; (ii) involvement of the upper trunk and neck; (iii) negative fungal staining of scales; (iv) no response to antifungal treatment; and (v) excellent response to minocycline.

This sensitivity thus appears as a good therapeutic test in case of diagnostic uncertainty [7, 8]. Recurrence is possible, however, despite a good initial response to treatment.

## 5. Conclusion

Confluent and reticulated papillomatosis of Gougerot-Carteaud is a special entity different from tinea versicolor which it simulates very often especially on black skin. Its sensitivity to oral treatment with tetracyclines (doxycycline or minocycline) is an important distinguishing criterion, useful for the diagnosis which should be evoked in front of these reticulated confluent papules.
